# Computationally Inexpensive Approach for Pitch Control of Offshore Wind Turbine on Barge Floating Platform

**DOI:** 10.1155/2013/357849

**Published:** 2013-12-22

**Authors:** Shan Zuo, Y. D. Song, Lei Wang, Qing-wang Song

**Affiliations:** ^1^Institute of Intelligent System and Renewable Energy Technology, University of Electronic Science and Technology of China, Chengdu 611731, China; ^2^Intelligent Systems and New Energy Technology Research Institute, Chongqing University, Chongqing 400044, China

## Abstract

Offshore floating wind turbine (OFWT) has gained increasing attention during the past decade because of the offshore high-quality wind power and complex load environment. The control system is a tradeoff between power tracking and fatigue load reduction in the above-rated wind speed area. In allusion to the external disturbances and uncertain system parameters of OFWT due to the proximity to load centers and strong wave coupling, this paper proposes a computationally inexpensive robust adaptive control approach with memory-based compensation for blade pitch control. The method is tested and compared with a baseline controller and a conventional individual blade pitch controller with the “NREL offshore 5 MW baseline wind turbine” being mounted on a barge platform run on FAST and Matlab/Simulink, operating in the above-rated condition. It is shown that the advanced control approach is not only robust to complex wind and wave disturbances but adaptive to varying and uncertain system parameters as well. The simulation results demonstrate that the proposed method performs better in reducing power fluctuations, fatigue loads and platform vibration as compared to the conventional individual blade pitch control.

## 1. Introduction

**Figure 1:**
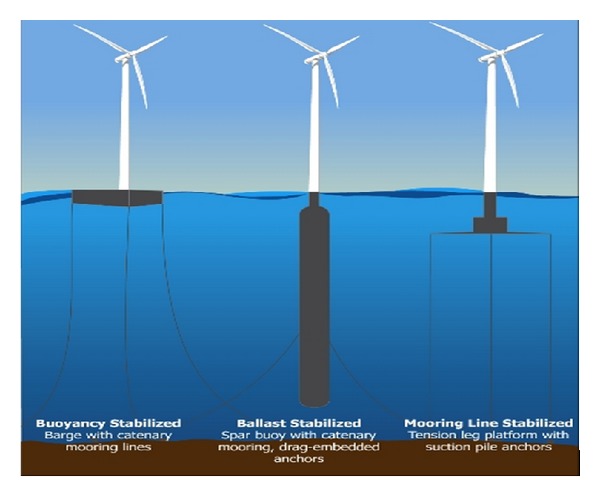
Floating offshore wind turbine concept. (Image from Wiki Commons.)

With the rapidly continuing development of wind energy all over the world, promising and reliable wind turbine concepts have been developed. Offshore floating wind turbine makes it possible to go further into water deeper than 60 m [1]. Figure 1 shows the three primary types of floating wind turbine: barge with catenary mooring lines, spar buoy with catenary mooring, and drag-embedded anchors and tension leg platform with suction pile anchors.

Control of offshore floating wind turbine is a relatively new yet challenging research area. The primary target for the control system of OFWT is to decrease output power fluctuations and minimize fatigue levels as well as platform motions simultaneously [2–5]. However, because of the external disturbances and uncertain system parameters of OFWT due to the much more complicated external load environment and strong wave coupling compared to the onshore wind turbine, an advanced robust adaptive control system is urgently needed to regulate power output and reduce fatigue loads.

There have been several recent achievements in this research area. Jonkman and Matha do a wide range of research on the three floating platforms with a baseline controller, respectively. However, the control objectives to regulate power output and reduce platform movements are found to essentially fight each other [6, 7]. The simulation results show some reduced platform movements and large increased output power fluctuations simultaneously. In order to avoid resonant pitch movement and improve fatigue life, Nielsen et al. establish an estimator based controller [8, 9]. Results show improvements in fatigue level, but power fluctuation increased at the same time. Namik and Stol utilize the barge platform model and present an advanced method with a linear quadratic regulator (LQR) controller to improve power fluctuation and platform movement [10, 11]. In the previous study, they apply the collective blade pitch control (CBP) to control power output and platform movement simultaneously. Results show impressive improvement as the power fluctuation is reduced by nearly 45%, while the platform movement is reduced by about 12%. However, the tower side-side moment increases up to 20%. Continuing the work done by Namik and Stol [12, 13], they develop a state space feedback controller with individual blade pitch control approach (IBP). By controlling each blade pitch angle, the aerodynamic loads are added to the previous symmetric loads caused by CBP to help control the platform movement. Simulation results show successful ability to reduce the power fluctuation by approximately 25%, the platform movement up to 30%, and the tower fore-aft loads by nearly 20%, respectively, while the blade root flapwise moment increases nearly 10%, which is created by the nonuniform thrust moment on the blades. Later on, Namik and Stol present a disturbance accommodating controller (DAC) to reduce the external load disturbance based on the previous SSFC and then apply the 5 MW OFWT model installed on the three types of floating platforms for performance comparison [14].

In this study, to address the challenge that the system parameters of OFWT are varying and uncertain due to the complex external wind and wave disturbances, an advanced control scheme is presented for blade pitch angle compensation. The added control approach is based on generalizing and utilizing previous system responses and control experience, which does not need any specified information about the nonlinear external wind and wave disturbances, and there is no need for linearization or approximation to the system dynamics. The advanced controller, consisting of a SSFC with a DAC and an additional IBP action via memory-based compensation, is presented and mounted on the barge platform for performance comparison with the baseline controller and the conventional DAC in above-rated wind speed region.

Section 2 briefly presents the wind turbine model and the barge floating platform used in this paper.  Section 3 describes the three implemented controllers: the baseline controller, an IBP SSFC with DAC, and an advanced memory-based individual blade pitch controller.  Section 4 shows the simulation and results, where the performances of the three controllers are compared with each other on barge floating platform. Eventually, conclusions are reported in Section 5.

## 2. Wind Turbine and Platform Models

### 2.1. 5 MW Offshore Wind Turbine Model

In this paper, the specified wind turbine model used for analysis is the “NREL offshore 5 MW baseline wind turbine” [15]. The physical properties of this wind turbine are listed in Table 1. This wind turbine is mounted on the barge floating platform.

### 2.2. Floating Platform

The barge platform is modeled for the support structure. The physical properties of the barge platform used in this paper are summarized in Table 2 [16]. The barge is a rectangular platform with eight catenary mooring lines.

## 3. Control Development

This section gives the detailed information about the three controllers simulated in the analysis.

### 3.1. The Baseline Controller

The baseline controller is built on the best performance controller presented by Jonkman in his previous research to alleviate platform movement on the barge floating platform [17]. The baseline controller includes two separate controllers: a generator torque controller and a collective blade pitch (CBP) controller.

In the above-rated wind speed condition, the purpose is to modulate output power to the rated value *P*_Rated_, and the generator torque *T*_Gen_ is inversely proportional to the generator speed *Ω*_Gen_, given by

(1) $$\begin{array}{c} T_{\text{Gen}}=\frac{P_{\text{Rated}}}{\eta_{\text{Gen}}\Omega_{\text{Gen}}}. \end{array}$$

The gain scheduled PI pitch controller is given by

(2) $$\begin{array}{c} \theta \left(t\right)=K_{p}e\left(t\right)+K_{i}\overset{t}{\underset{0}{\int }}e\left(\tau \right)d\tau , \end{array}$$

where *θ*(*t*) is the commanded blade pitch angle and *K*_*p*_ and *K*_*i*_ are the scheduled proportional and integral gain, respectively.

This controller has been used as a baseline controller to which the performance of the modified controllers could be compared because of its robustness in performance to model uncertainties.

### 3.2. Individual Blade Pitch Control with Disturbance Accommodation

This controller is simply the state space feedback controller (SSFC) with an additional disturbance accommodating controller (DAC) to minimize the influence of persistent disturbances like wind speed fluctuations and turbulent waves that affect a dynamic system [14].

The linearized periodic state space model is expressed as

(3) $$\begin{array}{c} \Delta \dot{\tilde{x}}=A\left(t\right)\Delta \tilde{x}+B\left(t\right)\Delta \tilde{u},\\ \tilde{x}\;=\Delta \tilde{x}+{\tilde{x}}^{\text{op}},\\ \tilde{u}=\Delta \tilde{u}+{\tilde{u}}^{\text{op}}, \end{array}$$

where $\tilde{x}$ and $\tilde{u}$ are the state and control input vectors, respectively; *A*(*t*) and *B*(*t*) are the periodic state and control gain matrices, respectively; ${\tilde{x}}^{\text{op}}$ and ${\tilde{u}}^{\text{op}}$ are the states and control operating points, respectively. The symbol Δ denotes perturbations about the linearization point.

The LQR is implemented in the full state feedback form, where all states could be measured and fed back to the controller.

The periodic SSFC control law is established as follows:

(4) $$\begin{array}{c} \tilde{u}=-K_{\text{LQR}}\Delta \tilde{x}+{\tilde{u}}^{\text{op}}, \end{array}$$

where *K*_LQR_ is the SSFC optimal gain matrix that minimizes the LQR cost function by solving the algebraic Riccati differential equation with respect to performance objectives, given by

(5) $$\begin{array}{c} J=\overset{\infty }{\underset{0}{\int }}\left(x^{T}Qx+u^{T}Ru\right)dt, \end{array}$$

where *Q* is positive semidefinite and *R* is positive definite.

The disturbance estimator model is given by

(6) $$\begin{array}{c} {\tilde{\dot{\underline{w}}}}_{\text{NR}}=\left({\overset{-}{A}}_{\text{NR}}-K_{e,\text{NR}}{\overset{-}{C}}_{\text{NR}}\right){\tilde{\underline{w}}}_{\text{NR}}+E_{\text{NR}}{\tilde{v}}_{\text{NR}}, \end{array}$$

where the subscript NR denotes the nonrotating frame of reference, ${\tilde{v}}_{\text{NR}}={\left[\begin{array}{cc} \Delta {\tilde{u}}_{\text{NR}} & \Delta {\tilde{x}}_{\text{NR}} \end{array}\right]}^{T}$contains the MBC transformed inputs, ${\overset{-}{A}}_{\text{NR}}=\left[\begin{array}{cc} A_{\text{NR}} & B_{d,\text{NR}}\Theta \\ 0 & F \end{array}\right]$, ${\overset{-}{B}}_{\text{NR}}=\left[\begin{array}{c} B_{\text{NR}}\\ 0 \end{array}\right]$, ${\overset{-}{C}}_{\text{NR}}=\left[\begin{array}{cc} C_{\text{NR}} & 0 \end{array}\right]$, $E_{\text{NR}}=\left[\begin{array}{cc} {\overset{-}{B}}_{\text{NR}} & K_{e,\text{NR}} \end{array}\right]$, and *K*_*e*,NR_ is the estimator gain [13].

The DAC control model is given by

(7) $$\begin{array}{c} \Delta {\tilde{u}}_{\text{NR}}=-K_{\text{NR}}\Delta {\tilde{x}}_{\text{NR}}+G_{d,\text{NR}}\tilde{\underline{z}}, \end{array}$$

where $\tilde{\underline{z}}$ is the estimated disturbance states vector and NR denotes a nonrotating frame, consisting of a state regulation part with FSFC and a disturbance minimizing part. *G*_*d*,NR_ is the disturbance minimization gain which is calculated based on the linearized system properties and the estimated disturbance waveforms.

**Figure 2:**
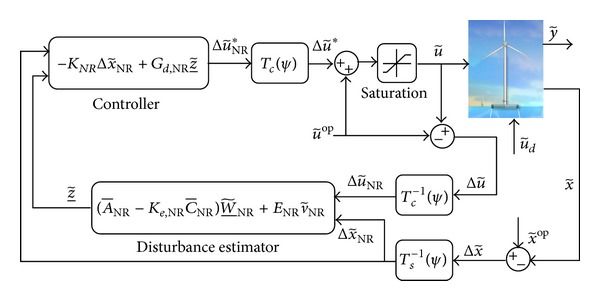
SSFC with DAC implementation for offshore wind turbine.

Figure 2 depicts the overall control block of DAC after multiblade coordinate (MBC) transformation with SSFC for the state regulation part of the controller. $\Delta {\tilde{u}}^{*}$ depicts the commanded actuator input vector. MBC transformation matrices *T*_*c*_(*ψ*), *T*_*c*_^−1^(*ψ*), and *T*_*s*_^−1^(*ψ*) transform the corresponding input vectors to the nonrotating frame of reference and vice versa; for detailed derivation of these matrices, please refer to [12].

### 3.3. Individual Blade Pitch Controller with Memory-Based Pitch Compensation

**Figure 3:**
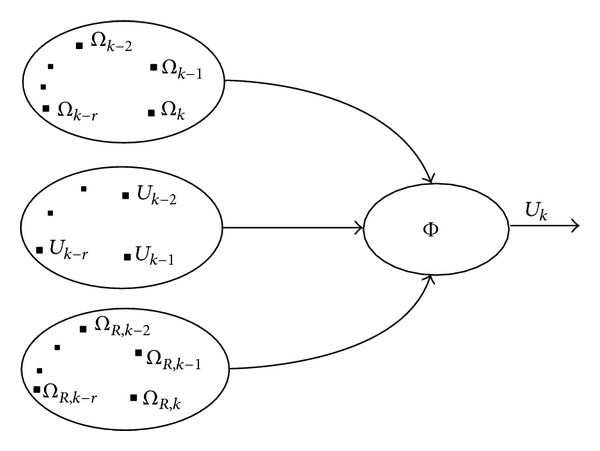
Memory-based pitch controller.

In this controller, a pitch angle adjustment through memory-based control approach is added to the SSFC discussed in the previous section. The advanced control approach utilizes some collected system information, like the latest tracking error, current tracking error, and the past control experience to straightly amend the current control command rather than the control gains [18, 19]. Therefore, the advanced control approach does not demand any accurate information about the nonlinear external wind and wave disturbances, and there is no need for approximation or linearization to the nonlinear system of the OFWT for control design. The control algorithm is conceptually demonstrated in Figure 3.

In this study, we only use the first order form of memory-based pitch control (MBPC), given by

(8) $$\begin{array}{c} U_{k}=\Phi \left(\delta ,\lambda_{0}U_{k-1}+\lambda_{1}e_{k}+\lambda_{2}e_{k-1}\right),\\ \Phi \left(\delta ,z\right)=k\frac{2}{\delta }\left(\frac{1-e^{-\delta z}}{1+e^{-\delta z}}\right),\;\delta >0,\;\;k>0, \end{array}$$

where *e*_*k*_ = *Ω*_*k*_ − *Ω*_*R*,*k*_ stands for the current generator speed tracking error, *e*_*k*−1_ = *Ω*_*k*−1_ − *Ω*_*R*,*k*−1_ is the one step back generator speed tracking error, *U*_*k*−1_ represents the previous control history experience,  Φ(·) is the related mapping function, *δ* is the control parameter, and *λ*_*j*_(*j* = 0,1, 2) are memory-based coefficients. As is shown in Figure 3, we only need the memory-based information above to construct the advanced controller.

**Remark 1**

Note that the proposed mapping function Φ(·) has the following features:

- |Φ(*δ*, *z*)| ≤ 2*k*/*δ*,
- lim_*δ*→0_Φ(*z*, *δ*) → *kz*.

Therefore, it can be established that Φ(·) approximates a linear function, the value of which can be confined within certain range by choosing suitable values of *δ* and *k*.

To address the challenge that the system parameters of OFWT are varying and uncertain due to the complex external wind and wave disturbances, we present an advanced control scheme for blade pitch angle compensation, which is implemented as

(9) $$\begin{array}{c} \theta_{k}^{cm}=\lambda_{0}\theta_{k-1}^{cm}+\lambda_{1}e_{k}+\lambda_{2}e_{k-1}. \end{array}$$

The equation of generator speed in above-rated condition is of the following form:

(10) $$\begin{array}{c} \dot{\Omega }=\left(\frac{1}{J}\right)\left(\left(\frac{1}{2}\right)\rho \pi R^{3}\frac{C_{p}\left(\lambda ,\theta^{cm}\right)}{\lambda }{\left(u^{cm}\right)}^{2}-\left(T_{g}+K_{t}\Omega \right)\right). \end{array}$$

In a specific operating point, the nonlinear equation in (10) can be linearized as

(11) $$\begin{array}{c} \dot{\Omega }=\left(\frac{1}{J}\right)\chi_{0}\Omega +\psi_{0}u^{cm}+\upsilon_{0}\theta^{cm}, \end{array}$$

where *χ*_0_, *ψ*_0_, and *υ*_0_ are system parameters in accordance with the different operating point.

Obviously, the system parameters will no longer be constant when the OFWT operates at varying speed condition. In addition, the linear approximation error may become vital due to the changes in operating point. Therefore, the advanced system model is implemented as

(12) Ω˙=1J(χ0+Δχ)Ω+(υ0+Δυ)θcm+ψucm+ζ(Ω,ucm,θcm),

where *ζ*(*Ω*, *u* ^*cm*^, *θ* ^*cm*^) represents the effect of the linearization. For the control purpose, we express (12) as

(13a) $$\begin{array}{c} \dot{\Omega }=\left(\frac{1}{J}\right)\left(\chi_{0}\Omega +\upsilon_{0}\theta^{cm}\right)+\Gamma \left(\psi ,\Omega ,\theta^{cm},u^{cm}\right), \end{array}$$

(13b) $$\begin{array}{c} \Gamma \left(\cdot \right)=\left(\frac{1}{J}\right)\left(\Delta \chi \Omega +\Delta \upsilon \theta^{cm}+\psi u^{cm}\right)+\zeta \left(\Omega ,u^{cm},\theta^{cm}\right), \end{array}$$

where Γ(·) stands for the system uncertainty due to the varying operating point. The accurate expression for Γ(·) is normally unavailable. However, the system uncertainty for a practical offshore wind turbine does not change all of a sudden. So we assume that

(14) $$\begin{array}{c} \underset{t\geq 0}{\max}\left|\frac{d\Gamma \left(\cdot \right)}{dt}\right|\leq c_{0}<\infty , \end{array}$$

which means that the variation rate of Γ(·) is limited.

The overall memory-based control is generated through

(15a) $$\begin{array}{c} \theta_{1}^{cm}=\theta^{c}+\theta^{m}, \end{array}$$

where *θ* ^*c*^ represents preliminary compensation and *θ* ^*m*^ denotes memory-based compensation. Making use of the available information about the OFWT, we construct the preliminary compensation expressed as

(15b) $$\begin{array}{c} \theta^{c}=\left(\frac{J}{\upsilon_{0}}\right)\left(-\left(\frac{\chi_{0}}{J}\right)\Omega -k_{0}e+{\dot{\Omega }}_{R}\right), \end{array}$$

where *e* = *Ω* − *Ω*_*R*_ is the generator speed tracking error and *k*_0_ > 0 is the system design parameter.

In this study, the value of the rated generator speed *Ω*_*R*_ is a plain linear equation of the platform pitch velocity ${\dot{\theta }}_{\text{plat}}$, given by

(16) $$\begin{array}{c} \Omega_{R}=1173.7\;\text{rpm}\cdot \left(1+k\cdot {\dot{\theta }}_{\text{plat}}\right), \end{array}$$

where the value of 1173.7 is the rated generator speed for the specified wind turbine used in our study. The slope *k* in the above equation is negative for a positive $\dot{\theta }$, which denotes the platform pitching downwind. Lackner has tested the value of *k* = −0.0375 in his previous work [20, 21]. Multiplying two sides of the above equation by the rated generator torque *T*_*R*_ = 43093.55 Nm, we find that the rated power of the wind turbine also varies according to $\dot{\theta }$, the value of which equals 5 MW when $\dot{\theta }$ is zero. This control scheme is called “variable power pitch control” (VPPC) in Matthew's previous work. He also tested and demonstrated the impressive results with effective reduction in the platform movement and tower fatigue loads with little influence on output power.

Substituting (15a) and (15b) into (13a) and (13b), we get

(17) $$\begin{array}{c} \dot{e}=-k_{0}e+\left(\frac{\upsilon_{0}}{J}\right)\theta^{m}+\Gamma \left(\cdot \right). \end{array}$$

In order to incorporate the memorized information, we construct the discrete form of (17) by Euler formula:

(18) $$\begin{array}{c} e_{k+1}=\left(1-k_{0}T\right)e_{k}+T\left(\left(\frac{\upsilon_{0}}{J}\right)\theta_{k}^{m}+\Gamma_{k}\left(\cdot \right)\right).\;\; \end{array}$$

To utilize the previous control history, we execute one step backward time shifting in (18), which leads to

(19) $$\begin{array}{c} e_{k}=\left(1-k_{0}T\right)e_{k-1}+T\left(\left(\frac{\upsilon_{0}}{J}\right)\theta_{k-1}^{m}+\Gamma_{k-1}\left(\cdot \right)\right). \end{array}$$

Subtracting (19) from (18), we get

(20) ek+1=(2−k0T)ek+(k0T−1)ek−1+T(Γk(·)−Γk−1(·))  +T(υ0J)(θkm−θk−1m).

By designating the memory coefficients *w*_0_, *w*_1_, and *w*_2_ we get

(21) $$\begin{array}{c} w_{0}=1,\;\;w_{1}=\left(k_{0}-\frac{2}{T}\right)\left(\frac{J}{\upsilon_{0}}\right),\\ w_{2}=\left(\frac{1}{T}-k_{0}\right)\left(\frac{J}{\upsilon_{0}}\right). \end{array}$$

Then the memory-based control part (9) simplifies (20) to

(22) $$\begin{array}{c} e_{k+1}=T\left(\Gamma_{k}\left(\cdot \right)-\Gamma_{k-1}\left(\cdot \right)\right). \end{array}$$

Incorporate (14) with (22), we get

(23) $$\begin{array}{c} \left|e_{k+1}\right|=T\left|\left(\Gamma_{k}\left(\cdot \right)-\Gamma_{k-1}\left(\cdot \right)\right)\right|\leq T^{2}\underset{t\geq 0}{\max}\left|\frac{d\Gamma \left(\cdot \right)}{dt}\right|\leq T^{2}c_{0}. \end{array}$$

**Figure 4:**
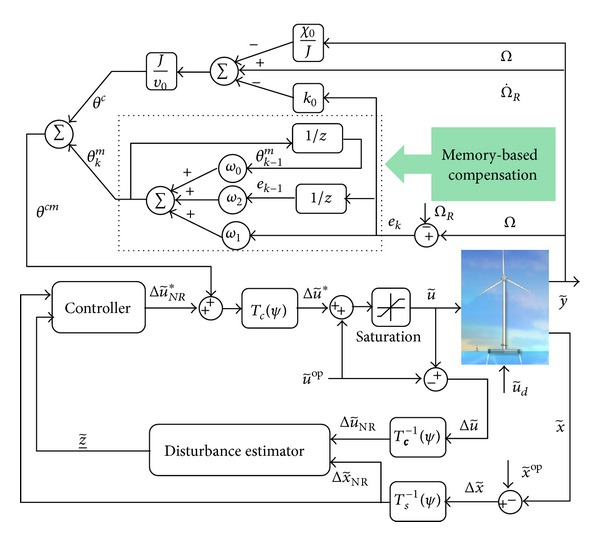
Block diagram of the overall control scheme.

Note that, by choosing  *T*  properly, fairly good tracking accuracy can be obtained. The overall control scheme is presented in Figure 4.

## 4. Simulation and Results

In this section, the “NREL offshore 5 MW baseline wind turbine” installed on a floating barge is tested and simulated with the FAST and MATLAB/Simulink under average 11.4 m/s turbulence wind speed specified by the National Renewable Energy Laboratory (NREL) [22].

In order to compare the three different types of controllers described in Section 3, nine performance measures are simulated: power and generator speed regulation, blade root flapwise moment and edgewise moment, tower base pitching moment and yaw moment, platform rolling, pitching, and yawing motions. Two kinds of calculating methods are used to calculate the nine performance measures, which are root mean square error (RMSE) and fatigue damage equivalent load (DEL). The DEL method uses simple rain flow counting algorithm [23].

**Figure 5:**
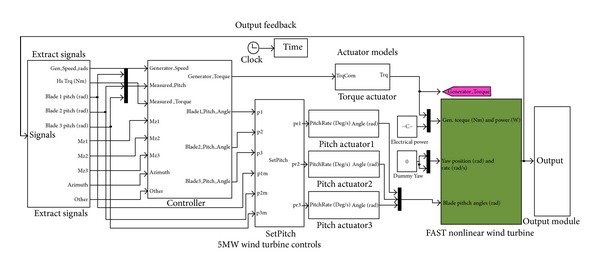
Overall simulation module of the controller implementation.

Figure 5 shows the overall simulation module of the controller implementation.

**Figure 6:**
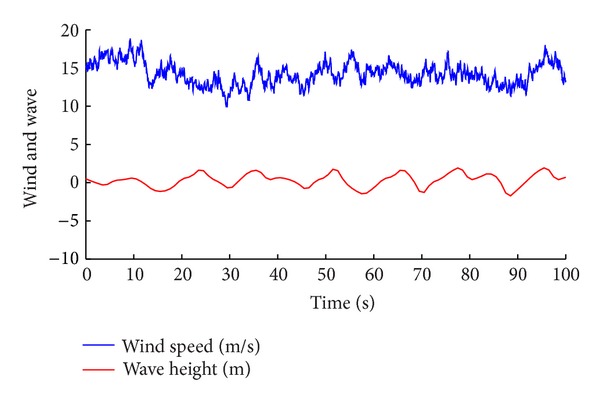
Wind and wave conditions.

Figure 6 shows the turbulence wind and wave conditions. The three types of controllers described in Section 3 are simulated and compared with each other based on the performance of power tracking and fatigue level reduction on the barge platform separately.

**Figure 7:**
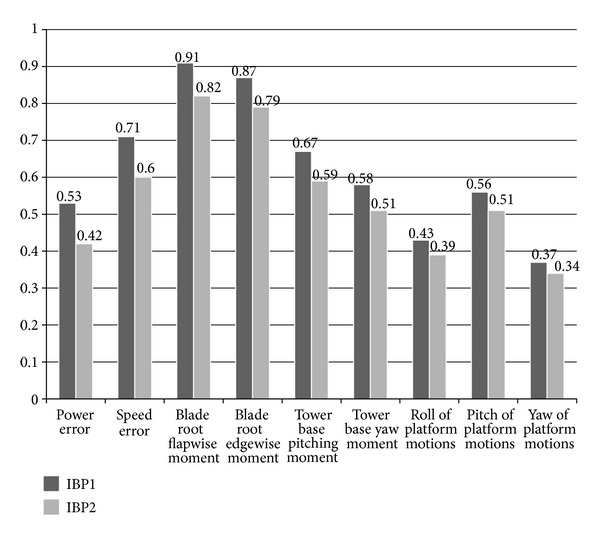
Comparison of averaged DLC for the barge platform with the baseline controller.

Figure 7 depicts the overall normalized simulation results using the NREL offshore 5 MW wind turbine model installed on a barge platform with incident wind and wave conditions (IBP1 refers to the conventional state space feedback controller and IBP2 refers to the advanced memory-based pitch controller). Normalized results indicate the following compared to the baseline controller.

- Output power: the SSFC is able to regulate power fluctuations by 47%, while the advanced SSFC with memory-based pitch compensation achieved significant 68% reduction.
- Wind turbine fatigue DEL: blade root flapwise moment, edgewise moment, tower base pitching moment, yaw moment are reduced by up to 9%, 13%, 33%, and 42% by the SSFC, respectively, while the advanced SSFC with MBPC decreased fatigue loading by 18%, 21%, 41%, and 49%, respectively.
- Platform motions: the conventional SSFC achieved up to 57%, 44%, and 63% reduction in platform rolling, pitching, and yawing, respectively. The advanced SSFC with MBPC reduced platform motions by 61%, 49%, and 66%.

**Figure 8:**
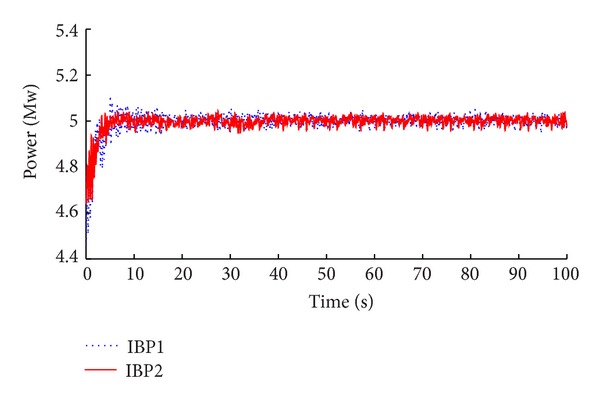
Comparison of power tracking.

**Figure 9:**
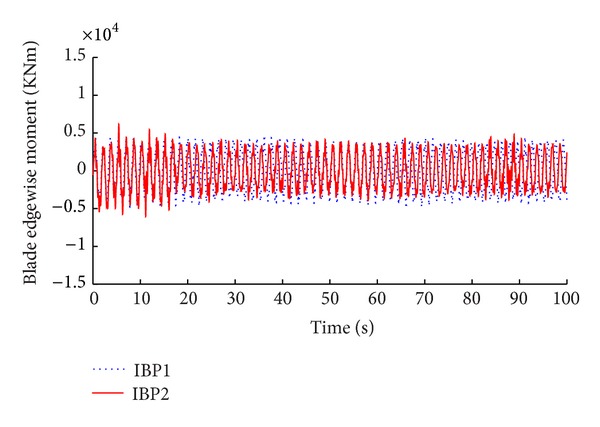
Comparison of blade edgewise moment.

**Figure 10:**
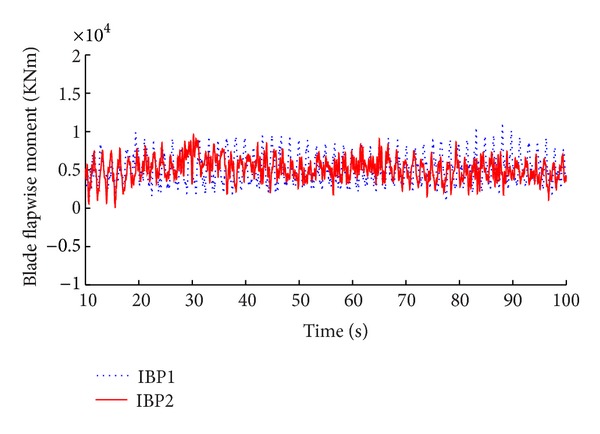
Comparison of blade flapwise moment.

**Figure 11:**
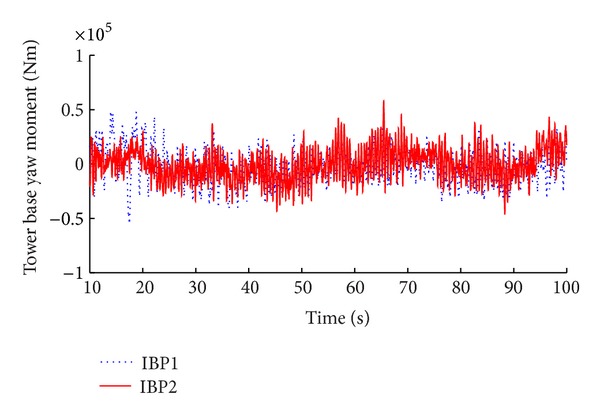
Comparison of tower base yaw moment.

The simulation results of the advanced IBP controller with memory-based compensation are demonstrated in Figures 8, 9, 10, and 11 compared with the same performance of the conventional state space feedback control on the basis of power regulation and fatigue level alleviation.

## 5. Conclusions

This paper focuses on the variable blade pitch control of OFWT for power tracking and fatigue load reduction on a barge platform. In allusion to the external disturbances and uncertain system parameters of OFWT due to the much more complicated external load environment and strong wave coupling compared to the onshore wind turbine, a computationally inexpensive robust adaptive control approach with memory-based compensation for blade pitch control is presented. Three different controllers are implemented on a barge platform for performance comparison: a baseline controller, a conventional state space feedback controller (SSFC), and an SSFC with memory-based pitch compensation. The simulations are tested on the basis of the IEC-61400-3 standard for offshore floating wind turbine design.

According to the averaged simulation results, the memory-based pitch control approach is not only robust to complex wind and wave disturbances but adaptive to varying and uncertain system parameters as well. As a result, the advanced controller shows a better performance in reducing power fluctuations, fatigue loads, and platform vibrations, and therefore, is more suitable for large offshore wind turbines.

## Figures and Tables

**Table 1:** NREL 5 MW turbine model properties.

Power rating	5 MW
Rotor orientation	Upwind
Control	Variable speed, variable pitch, and active yaw
Rotor, hub diameter	126 m, 3 m
Hub height	90 m
Rated rotor, generator speed	12.1 rpm, 1173.7 rpm
Blade operation	Pitch to feather
Maximum blade pitch rate	8°/s
Rated generator torque	43,093 Nm
Maximum generator torque	47,402 Nm

Using the turbine model data from [15].

**Table 2:** Physical properties of the barge platform.

Width × length × height	40 m × 40 m × 10 m
Draft	4 m
Platform mass	5,452,330 kg
Water depth	150 m
Platform pitch natural frequency	0.078 Hz

Using the barge platform data from [16].

## Appendix

Equation (A.1) lists all the equations applied for MBC transformation [12]:

(A.1) $$\begin{array}{c} T_{c}={\left[\begin{array}{cccc} I_{Fc\times Fc} & & & \\ & \tilde{t} & & \\ & & \ddots & \\ & & & \tilde{t} \end{array}\right]}_{m\times m},\;\;T_{s}={\left[\begin{array}{cc} T_{1} & 0\\ \Omega T_{2} & T_{1} \end{array}\right]}_{n\times n},\\ T_{1}={\left[\begin{array}{cccc} I_{nF\times nF} & & & \\ & \tilde{t} & & \\ & & \ddots & \\ & & & \tilde{t} \end{array}\right]}_{n/2\times n/2},\\ T_{2}={\left[\begin{array}{cccc} 0_{nF\times nF} & & & \\ & {\tilde{t}}_{2} & & \\ & & \ddots & \\ & & & {\tilde{t}}_{2} \end{array}\right]}_{n/2\times n/2},\\ {\tilde{t}}_{2}=\left[\begin{array}{ccc} 1 & -\sin\varphi_{1} & \cos\varphi_{1}\\ 1 & -\sin\varphi_{2} & \cos\varphi_{2}\\ 1 & -\sin\varphi_{3} & \cos\varphi_{3} \end{array}\right]\;\;\tilde{t}=\left[\begin{array}{ccc} 1 & \cos\varphi_{1} & \sin\varphi_{1}\\ 1 & \cos\varphi_{2} & \sin\varphi_{2}\\ 1 & \cos\varphi_{3} & \sin\varphi_{3} \end{array}\right]. \end{array}$$
